# Lamotrigine, Contraceptives, and Psychiatry: A Narrative Review

**DOI:** 10.7759/cureus.100392

**Published:** 2025-12-30

**Authors:** Inês Costa, Daniel Esteves-Sousa

**Affiliations:** 1 Department of Psychiatry, Unidade Local de Saúde da Lezíria, Santarém, PRT; 2 Department of Psychiatry, Unidade Local de Saúde de Lisboa Ocidental, Lisbon, PRT

**Keywords:** contraceptives, estrogens, lamotrigine, progestogens, psychiatric disorders, reproductive age

## Abstract

Lamotrigine is widely used in neurology and psychiatry, particularly in epilepsy and mood disorders, due to its favorable efficacy and safety profile. However, clinically relevant pharmacokinetic interactions between lamotrigine and hormonal contraceptives have been increasingly recognized, raising concerns regarding therapeutic stability and reproductive safety in women of reproductive age. This narrative review examines the bidirectional interactions between lamotrigine and hormonal contraceptive methods, focusing on their pharmacokinetic mechanisms, clinical consequences, and implications for prescribing practice. Relevant studies were identified and narratively synthesized to investigate the bidirectional interactions between lamotrigine and hormonal contraceptive methods, focusing on their pharmacokinetic mechanisms, clinical consequences, and implications for prescribing practice. Available evidence consistently demonstrates that estrogen-containing contraceptives significantly reduce lamotrigine plasma concentrations through induction of hepatic glucuronidation, leading to substantial interindividual and intracycle variability. Cyclic contraceptive regimens further contribute to marked fluctuations in lamotrigine levels, with potential risks of subtherapeutic exposure during active hormone phases and toxicity during hormone-free intervals. In contrast, lamotrigine appears to exert minimal and clinically inconsistent effects on contraceptive efficacy, although hormonal fluctuations and breakthrough bleeding may occur. Progestin-only and non-hormonal contraceptive methods demonstrate more favorable pharmacokinetic profiles and are associated with greater therapeutic stability. Although most data derive from epilepsy populations, these interactions are also relevant in psychiatric practice, where lamotrigine is commonly prescribed for mood stabilization and where modest pharmacokinetic shifts may have a significant clinical impact. An understanding of these interactions is essential to avoid misinterpretation of symptom recurrence, prevent adverse effects, and support informed contraceptive counseling. An individualized, multidisciplinary approach is recommended to optimize both psychopharmacological treatment and reproductive care.

## Introduction and background

Lamotrigine is a second-generation antiepileptic drug extensively used in the treatment of epilepsy and increasingly prescribed in psychiatry, particularly for bipolar depression and maintenance therapy in bipolar disorder. Its mechanism of action primarily involves inhibition of voltage-gated sodium channels and modulation of glutamatergic neurotransmission, contributing to both anticonvulsant and mood-stabilizing effects. The widespread adoption of lamotrigine in women of reproductive age has been largely driven by its favorable safety profile compared with older antiepileptic drugs, especially regarding teratogenic risk and cognitive adverse effects [1-3].

Antiepileptic drugs are well known for their potential to cause clinically significant drug-drug interactions, particularly through induction or inhibition of hepatic metabolic pathways. Classical enzyme-inducing antiepileptic drugs such as carbamazepine, phenytoin, phenobarbital, and topiramate can reduce circulating levels of hormonal contraceptives, increasing the risk of unintended pregnancy [2,4,5]. In contrast, lamotrigine was initially considered pharmacokinetically neutral with respect to hormonal contraception. However, accumulating evidence over the past two decades has challenged this assumption, demonstrating that lamotrigine and hormonal contraceptives interact in a bidirectional and clinically relevant manner [1,6,7].

These interactions are particularly important in psychiatric populations. Women treated with lamotrigine for mood disorders often receive lower doses than those used in epilepsy, yet even modest fluctuations in plasma levels may have meaningful clinical consequences, including mood destabilization, pseudo-treatment resistance, or adverse effects related to toxicity [6,8]. At the same time, hormonal contraceptive choice is a critical aspect of care in this population, given the potential impact of unplanned pregnancy, medication discontinuation, and relapse of psychiatric illness.

Despite growing recognition of these issues, clinical awareness remains variable, and prescribing practices are often not systematically adapted to account for lamotrigine-contraceptive interactions [2,9].

This article is presented as a narrative review, aiming to synthesize available evidence on the pharmacokinetic mechanisms, clinical consequences, and practical management of interactions between lamotrigine and hormonal contraceptives, with a particular focus on their relevance to psychiatric practice.

Methods

A literature search was conducted using PubMed, employing combinations of the terms “lamotrigine”, “contraception”, “oral contraceptives”, and “antiepileptic drugs”. Articles published in English between 2003 and 2025 were considered eligible.

Seventeen original studies met the inclusion criteria and were included in the narrative synthesis. Original clinical studies, pharmacokinetic trials, and observational studies examining interactions between lamotrigine and contraceptive methods were included. Review articles, clinical guidelines, and regulatory documents were also examined to contextualize findings and inform clinical recommendations. 

No formal quality appraisal or risk-of-bias assessment was performed, and a systematic selection process was not applied. Given the heterogeneity of study designs, populations, and outcome measures, a quantitative meta-analysis was not feasible, and findings were therefore narratively synthesized.

## Review

Mechanistic insights

The interaction between lamotrigine and combined hormonal contraceptives (CHCs) is the most consistently documented and clinically relevant finding across the literature. Multiple pharmacokinetic studies demonstrate that CHCs containing ethinyl estradiol significantly reduce lamotrigine plasma concentrations, with reductions commonly exceeding 50% relative to baseline values [1,7,10,11].

This interaction is primarily mediated by estrogen-induced upregulation of hepatic uridine diphosphate-glucuronosyltransferase (UGT), particularly UGT1A4, the main enzyme responsible for lamotrigine metabolism [1,4,12]. Estrogens and lamotrigine share glucuronidation pathways, leading to accelerated lamotrigine clearance when both agents are co-administered. This mechanism also explains the well-established inhibitory effect of valproate on lamotrigine metabolism, as valproate acts as a UGT inhibitor [4,13].

Beyond overall reductions in lamotrigine levels, cyclic CHC regimens introduce additional complexity. During the hormone-free interval, typically lasting seven days, estrogen withdrawal results in the abrupt cessation of enzyme induction, leading to a rapid rise in lamotrigine concentrations. Several studies report increases ranging from 80% to nearly 100% relative to levels observed during active pill phases [1,7,11]. This fluctuation has been associated with an increased risk of lamotrigine-related adverse effects, including dizziness, ataxia, diplopia, and cutaneous reactions [3].

Clinical evidence: supportive and conflicting findings

Importantly, endogenous hormonal fluctuations across the natural menstrual cycle do not appear to produce clinically significant changes in lamotrigine pharmacokinetics, suggesting that the interaction is specific to exogenous estrogen exposure rather than physiological variations [11].

Although estrogen is considered the principal driver of this interaction, emerging evidence suggests that certain progestins may exert additive effects. Rauchenzauner et al. observed statistically significant lamotrigine level fluctuations in women using ethinyl estradiol combined with levonorgestrel or drospirenone, while combinations containing gestodene appeared to produce more stable lamotrigine concentrations [1]. However, these findings are derived from small samples, and their clinical relevance remains uncertain.

The reciprocal effect of lamotrigine on contraceptive hormone levels has been less consistently demonstrated. A prospective pharmacokinetic study reported modest reductions in progestin concentrations, accompanied by elevations in follicle-stimulating hormone (FSH) and luteinizing hormone (LH), as well as intermenstrual bleeding in approximately one-third of participants after lamotrigine initiation [7]. Notably, ovulation was not documented, and no clear evidence of reduced contraceptive efficacy was established.

Subsequent studies have not conclusively demonstrated a clinically meaningful increase in contraceptive failure attributable to lamotrigine alone [2,5,9]. It is also relevant that modern CHCs typically contain relatively low estrogen doses, often near the threshold required to suppress ovulation, which may independently contribute to breakthrough bleeding or perceived contraceptive instability [2].

Overall, current evidence does not support a robust association between lamotrigine use and contraceptive failure. Nevertheless, hormonal fluctuations and bleeding irregularities may complicate adherence and patient confidence, indirectly affecting contraceptive reliability.

CHCs

Data on non-oral CHCs remain limited. Small prospective and observational studies suggest that transdermal contraceptive patches and vaginal rings may interact with lamotrigine in a manner comparable to oral formulations, leading to reduced lamotrigine concentrations during active hormone exposure. The vaginal ring containing ethinyl estradiol and etonogestrel has been shown to significantly lower lamotrigine plasma levels, reinforcing the role of estrogen as the principal mediator of interaction [14].

Given the paucity of robust data, these formulations are generally approached with the same caution as oral CHCs when co-prescribed with lamotrigine.

Progestin-only contraceptives (POCs)

POCs are widely considered a safer alternative for women treated with lamotrigine, although evidence is not entirely uniform. Most studies indicate minimal or no clinically relevant effect of progestin-only methods on lamotrigine metabolism [5,9,15]. However, isolated reports suggest that certain oral progestins, such as desogestrel, may modestly increase lamotrigine concentrations, possibly through competitive metabolic effects [9,11].

Non-oral formulations

Long-acting progestin-only methods appear to offer the most favorable pharmacokinetic profile. Injectable medroxyprogesterone acetate and levonorgestrel-releasing intrauterine devices demonstrate minimal interaction with lamotrigine, likely due to limited hepatic first-pass metabolism [9,10,16]. A prospective pilot study examining levonorgestrel intrauterine device use in women with epilepsy reported good tolerability and stable seizure control, indirectly supporting pharmacokinetic neutrality in this context [16].

Table 1 summarizes the key characteristics and main findings of the studies included in this narrative review, focusing on the bidirectional pharmacokinetic interactions between lamotrigine and hormonal contraceptive methods, including combined oral contraceptives, progestin-only formulations, and non-oral hormonal contraceptives. Reported outcomes include changes in lamotrigine plasma concentrations, hormonal levels, and relevant clinical implications

**Table 1:** Summary of included studies on lamotrigine-hormonal contraceptive interactions ACC, combined oral contraceptives; COC, combined oral contraceptive; EE, ethinyl estradiol; FSH, follicle-stimulating hormone; HC, hormonal contraception; IUD, intrauterine device; LH, luteinizing hormone; LTG, lamotrigine; POC, progestin-only contraceptive; UGT, uridine diphosphate–glucuronosyltransferase; US MEC, United States Medical Eligibility Criteria

Study (Author(s), Year)	Study Design	Population	Contraceptive Method	Main Findings
Rauchenzauner et al., 2020 [1]	Observational pharmacokinetic study	Women with epilepsy	Combined oral contraceptives (ethinyl estradiol + different progestins)	Ethinyl estradiol significantly reduced lamotrigine levels; magnitude of fluctuation varied according to progestin type
Wang et al., 2012 [2]	Retrospective population-based study	Women of reproductive age using antiepileptic drugs	Various hormonal contraceptives	High prevalence of co-prescription; frequent use of low-dose estrogen contraceptives
Suzuki et al., 2020 [3]	Prospective observational study	Patients treated with lamotrigine	Not contraceptive-specific	High early lamotrigine plasma concentrations associated with increased risk of cutaneous adverse reactions
Sabers, 2008 [4]	Narrative pharmacokinetic review	Women with epilepsy	Combined hormonal contraceptives	Estrogen induces lamotrigine metabolism via glucuronidation, reducing serum levels
Reimers et al., 2015 [5]	Mechanistic and clinical review	Women using antiepileptic drugs	Hormonal contraceptives	Estrogen-containing methods reduce lamotrigine levels; limited evidence for reverse interaction
Sawagashira et al., 2017 [6]	Case-based pharmacokinetic report	Patients with bipolar I disorder	Combined oral contraceptives	Lamotrigine level reduction associated with mood destabilization
Sidhu et al., 2006 [7]	Prospective pharmacokinetic trial	Healthy female volunteers	Combined oral contraceptives	Lamotrigine levels decreased during active pill phase and increased during hormone-free interval
Crawford, 2005 [8]	Clinical guideline	Women with epilepsy	Hormonal contraceptives	Recommends caution with hormonal contraceptives in patients receiving antiepileptic drugs
Gaffield et al., 2011 [9]	Systematic review	Women taking anticonvulsant therapy	Hormonal contraceptives	No consistent evidence that lamotrigine increases contraceptive failure
Maroney and Perumpail, 2023 [10]	Clinical narrative review	Psychiatric patients (mood disorders)	Oral contraceptives	Even modest lamotrigine level fluctuations may have significant psychiatric impact
Wegner et al., 2009 [11]	Pharmacokinetic study	Women treated with lamotrigine	Combined oral contraceptives	No significant lamotrigine fluctuations across natural menstrual cycle
Christensen et al., 2007 [12]	Double-blind, placebo-controlled trial	Women with epilepsy	Combined oral contraceptives	Oral contraceptives induced lamotrigine metabolism with >50% reduction in serum levels
Wegner et al., 2014 [13]	Observational cohort study	Women with epilepsy	Combined oral contraceptives + comedication	Effect of contraceptives on lamotrigine levels depended on concomitant antiepileptic drugs
King et al., 2020 [14]	Prospective pharmacokinetic study	Women with epilepsy	Vaginal contraceptive ring	Vaginal ring significantly reduced lamotrigine plasma concentrations
Schwenkhagen et al., 2008 [15]	Narrative clinical review	Women with epilepsy	Various contraceptive methods	Non-hormonal and progestin-only methods preferred with lamotrigine
Davis et al., 2016 [16]	Prospective pilot study	Women with epilepsy	Levonorgestrel intrauterine device	Good tolerability and stable clinical outcomes
Nguyen et al., 2024 [17]	National guideline (CDC, US MEC)	Women of reproductive age	All contraceptive methods	Estrogen-containing methods require caution when co-administered with lamotrigine

Discussion

The interaction between lamotrigine and hormonal contraception represents a paradigmatic example of a clinically meaningful pharmacokinetic interaction with implications that extend across neurology, psychiatry, and reproductive medicine. Unlike many drug-drug interactions that remain largely theoretical or of limited clinical relevance, the estrogen-lamotrigine interaction is supported by convergent evidence from randomized trials, pharmacokinetic studies, observational cohorts, and regulatory guidance, and has direct consequences for treatment efficacy, safety, and adherence [1,4,7,11,12].

From a mechanistic perspective, this interaction is primarily driven by estrogen-mediated induction of hepatic glucuronidation pathways, particularly uridine diphosphate-glucuronosyltransferase 1A4 (UGT1A4), which is the principal enzyme responsible for lamotrigine metabolism [1,4,12]. Induction of this pathway accelerates lamotrigine clearance, resulting in marked reductions in plasma concentrations that frequently exceed 50% of baseline levels [7,12]. Importantly, this effect can occur rapidly following initiation of estrogen-containing contraceptives, increasing the risk of subtherapeutic exposure and clinical destabilization if not anticipated.

Cyclic combined hormonal contraceptive regimens further amplify pharmacokinetic instability. During the hormone-free interval, withdrawal of estrogen leads to abrupt cessation of enzyme induction and a rebound increase in lamotrigine concentrations, often approaching or exceeding pre-contraceptive levels [1,7,11]. This repeated pattern of under- and overexposure within a single treatment cycle is particularly problematic for a drug with a relatively narrow therapeutic window. Clinically, such fluctuations have been associated with neurological adverse effects, including dizziness, ataxia, diplopia, and, in rare cases, serious cutaneous reactions, particularly during early titration or unsynchronized dose adjustments [3].

A clinically relevant distinction emerges when comparing exogenous versus endogenous hormonal influences. Studies examining lamotrigine pharmacokinetics across the natural menstrual cycle or following menopause consistently demonstrate minimal or no clinically meaningful variation [11]. This observation underscores that the interaction is specific to pharmacological estrogen exposure rather than physiological hormonal fluctuations, reinforcing the need for targeted contraceptive counseling rather than generalized concern regarding menstrual cycling.

Although estrogen is clearly the dominant driver of this interaction, the potential modulatory role of progestins remains incompletely defined. Observational data suggest that certain progestins, when combined with ethinyl estradiol, may influence the magnitude of lamotrigine fluctuations [1]. However, these findings are derived from small samples, lack consistent replication, and have not yet translated into reliable, progestin-specific prescribing recommendations.

The reciprocal question, whether lamotrigine compromises contraceptive efficacy, has been explored with less consistent findings. While pharmacokinetic studies demonstrate modest reductions in progestin levels and transient increases in follicle-stimulating hormone and luteinizing hormone following lamotrigine initiation [7], these changes have not been consistently associated with ovulation or confirmed contraceptive failure [2,5,9]. Breakthrough bleeding, although relatively common, likely reflects the low estrogen content of contemporary contraceptive formulations rather than true loss of contraceptive efficacy. Nevertheless, such bleeding may negatively impact adherence and patient confidence, indirectly increasing the risk of unintended pregnancy.

One of the most salient gaps identified in the literature is the relative paucity of data in psychiatric populations. Lamotrigine is widely prescribed for bipolar disorder and other mood disorders, often at lower doses than those used in epilepsy [6,10]. However, mood stabilization frequently depends on maintaining plasma concentrations within a relatively narrow effective range, and even modest pharmacokinetic perturbations may result in affective destabilization or apparent treatment resistance. Case reports describing mood worsening following initiation of estrogen-containing contraceptives in lamotrigine-treated patients highlight this vulnerability [6], but systematic studies in psychiatric cohorts remain lacking.

Polypharmacy introduces additional complexity. In bipolar disorder, lamotrigine is frequently co-prescribed with other mood stabilizers, particularly valproate, which inhibits lamotrigine metabolism [4,13]. In such cases, opposing pharmacokinetic forces, estrogen induction and valproate inhibition, may partially offset one another, potentially attenuating lamotrigine fluctuations. However, this balance is unpredictable and underscores the importance of individualized assessment rather than reliance on theoretical pharmacological counterweights.

From a population and public health standpoint, recent guidance reinforces the clinical relevance of these interactions. The 2024 update of the U.S. Medical Eligibility Criteria for Contraceptive Use explicitly recognizes the interaction between lamotrigine and estrogen-containing contraceptives, advising caution and individualized contraceptive selection in affected individuals [17]. Failure to anticipate pharmacokinetic interactions between lamotrigine and estrogen-containing contraceptives may result in clinically relevant consequences, including loss of seizure control due to reduced lamotrigine exposure during active hormone phases, dose-related adverse effects during periods of rapid plasma concentration increase, and inappropriate treatment escalation. These risks are particularly relevant in women of reproductive age with epilepsy, in whom estrogen-induced fluctuations in lamotrigine levels have been consistently shown to be clinically meaningful [1,7,11,12].

Collectively, available evidence supports a conceptualization of lamotrigine-contraceptive interactions as dynamic, clinically consequential processes shaped by formulation choice, dosing schedule, comedication, and patient-specific factors. Failure to recognize these interactions risks misattributing symptom recurrence to disease progression, nonadherence, or treatment failure, rather than to modifiable pharmacokinetic mechanisms.

In the absence of universally accepted, lamotrigine-specific contraceptive guidelines, clinical management must be guided by available evidence, regulatory recommendations, and expert consensus. When initiating lamotrigine in patients already using combined hormonal contraceptives, dose escalation may be required to achieve therapeutic efficacy, with maintenance doses sometimes needing to be increased up to twofold, guided by clinical response and tolerability. Conversely, discontinuation of estrogen-containing contraceptives necessitates cautious lamotrigine dose reduction to mitigate toxicity, with gradual tapering recommended to avoid abrupt plasma level increases [18,19].

Lamotrigine titration should ideally avoid periods immediately preceding or during the hormone-free interval of combined contraceptive regimens, as this phase is associated with the greatest pharmacokinetic instability [11,16]. Where combined hormonal contraceptives are maintained, continuous or extended-cycle formulations may reduce lamotrigine level oscillations and improve tolerability, provided no contraindications exist [10,11].

Progestin-only methods should be considered first-line contraceptive options in women receiving lamotrigine, given their more favorable interaction profile. Long-acting reversible contraceptives, including levonorgestrel-releasing intrauterine devices and injectable medroxyprogesterone acetate, demonstrate minimal interaction risk and are endorsed by both clinical reviews and recent eligibility criteria [9,10,16]. Non-hormonal methods, such as the copper intrauterine device, offer maximal pharmacokinetic neutrality and should be discussed whenever appropriate [1,15].

Regardless of the method selected, barrier contraception may be considered as an adjunct during periods of pharmacokinetic uncertainty or medication adjustment [9]. Shared decision-making involving psychiatry, neurology, and gynecology is strongly recommended, particularly in complex cases or when reproductive planning is a priority.

## Conclusions

Available evidence consistently demonstrates that estrogen-containing contraceptives exert a clinically significant inductive effect on lamotrigine metabolism, resulting in substantial reductions and cyclical fluctuations in serum lamotrigine concentrations. In contrast, lamotrigine appears to have minimal and clinically inconsistent effects on contraceptive efficacy. Among available contraceptive options, non-hormonal methods and long-acting progestin-only formulations provide the most stable pharmacokinetic profiles for individuals treated with lamotrigine.

Despite increasing awareness, the literature remains dominated by small studies and epilepsy-focused cohorts, limiting generalizability to psychiatric populations. Until further prospective data are available, clinicians should adopt an individualized, anticipatory approach that integrates pharmacokinetic principles, patient preferences, and interdisciplinary collaboration to ensure therapeutic stability while providing safe and effective reproductive care.
